# 2-Methyl-4-(naphthalen-2-yl)-3a-nitro-3,3a,4,9b-tetra­hydro-2*H*-spiro­[chromeno[3,4-*c*]pyrrole-1,3′-indolin]-2′-one

**DOI:** 10.1107/S160053681301533X

**Published:** 2013-06-08

**Authors:** Seenivasan Karthiga Devi, Thothadri Srinivasan, Jonnalagadda Naga Siva Rao, Raghavachary Raghunathan, Devadasan Velmurugan

**Affiliations:** a Centre of Advanced Study in Crystallography and Biophysics, University of Madras, Guindy Campus, Chennai 600 025, India; b Department of Organic Chemistry, University of Madras, Guindy Campus, Chennai 600 025, India

## Abstract

In the title compound, C_29_H_23_N_3_O_4_, the 2-methylpyrrolidine ring adopts a twist conformation on the N—C bond involving the spiro C atom, while the hydropyran ring adopts an envelope conformation with the methine C atom bonded to the O atom as the flap. The mean plane of the indoline-2-one ring system is almost perpendicular to the mean plane of the pyrrolidine ring, making a dihedral angle of 89.73 (8)°. The latter ring makes dihedral angles of 47.80 (8) with the naphthalene ring system and 32.38 (8)° with the hydropyran ring mean plane. There is an intra­molecular C-H⋯O hydrogen bond involving the indoline-2-one O atom. In the crystal, adjacent mol­ecules are linked *via* N—H⋯O hydrogen bonds, forming chains propagating along [100]. The chains are linked *via* weak C—H⋯O hydrogen bonds, forming two-dimensional networks, lying parallel to (101), and consolidated by C—H⋯π inter­actions.

## Related literature

For the biological importance of 4*H*-chromene derivatives, see: Cai (2007 ▶, 2008 ▶); Cai *et al.* (2006 ▶); Gabor (1988 ▶); Brooks (1998 ▶); Valenti *et al.* (1993 ▶); Hyana & Saimoto (1987 ▶); Tang *et al.* (2007 ▶). For applications of indoline-2-one and its derivatives as precursors in the synthesis of pharmaceuticals, see: Colgan *et al.* (1996 ▶).
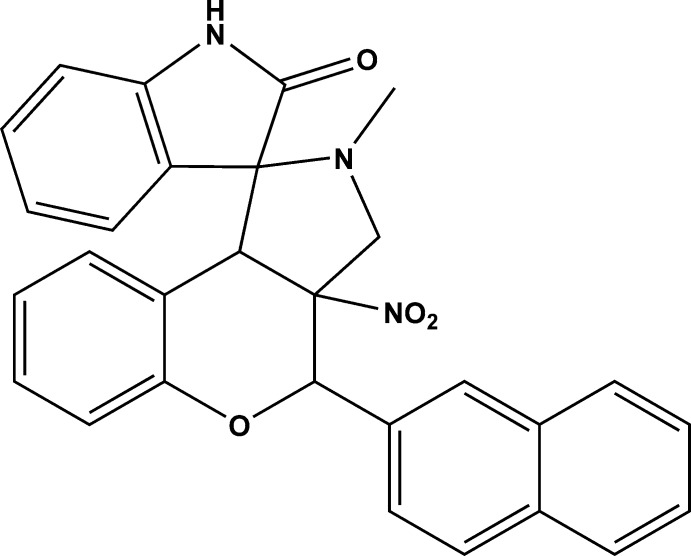


## Experimental

### 

#### Crystal data

C_29_H_23_N_3_O_4_
*M* *_r_* = 477.50
Monoclinic, 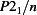

*a* = 9.4359 (6) Å
*b* = 16.5086 (11) Å
*c* = 15.1964 (10) Å
β = 96.363 (4)°
*V* = 2352.6 (3) Å^3^
*Z* = 4
Mo *K*α radiation
μ = 0.09 mm^−1^
*T* = 293 K
0.30 × 0.25 × 0.20 mm

#### Data collection

Bruker SMART APEXII area-detector diffractometer
Absorption correction: multi-scan (*SADABS*; Bruker, 2008 ▶) *T*_min_ = 0.973, *T*_max_ = 0.982
22349 measured reflections
5856 independent reflections
3862 reflections with *I* > 2σ(*I*)
*R*_int_ = 0.035

#### Refinement

*R*[*F* ^2^ > 2σ(*F* ^2^)] = 0.048
*wR*(*F* ^2^) = 0.141
*S* = 1.03
5856 reflections
329 parameters
1 restraint
H atoms treated by a mixture of independent and constrained refinement
Δρ_max_ = 0.27 e Å^−3^
Δρ_min_ = −0.18 e Å^−3^

### 

Data collection: *APEX2* (Bruker, 2008 ▶); cell refinement: *SAINT* (Bruker, 2008 ▶); data reduction: *SAINT*; program(s) used to solve structure: *SHELXS97* (Sheldrick, 2008 ▶); program(s) used to refine structure: *SHELXL97* (Sheldrick, 2008 ▶); molecular graphics: *ORTEP-3 for Windows* (Farrugia, 2012 ▶); software used to prepare material for publication: *SHELXL97* and *PLATON* (Spek, 2009 ▶).

## Supplementary Material

Crystal structure: contains datablock(s) global, I. DOI: 10.1107/S160053681301533X/su2607sup1.cif

Structure factors: contains datablock(s) I. DOI: 10.1107/S160053681301533X/su2607Isup2.hkl

Supplementary material file. DOI: 10.1107/S160053681301533X/su2607Isup3.cml

Additional supplementary materials:  crystallographic information; 3D view; checkCIF report

## Figures and Tables

**Table 1:** Hydrogen-bond geometry (Å, °) *Cg*1 is the centroid of the C10–C14/C19 ring.

*D*—H⋯*A*	*D*—H	H⋯*A*	*D*⋯*A*	*D*—H⋯*A*
C9—H9⋯O4	0.98	2.44	3.250 (2)	140
N3—H3*A*⋯O3^i^	0.87 (2)	2.52 (2)	3.220 (2)	138 (2)
C2—H2⋯O3^ii^	0.93	2.58	3.156 (2)	121
C3—H3⋯*Cg*1^ii^	0.93	2.57	3.473 (2)	164

Symmetry codes: (i) 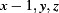; (ii) 
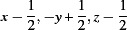
.

**Fig. 1.**
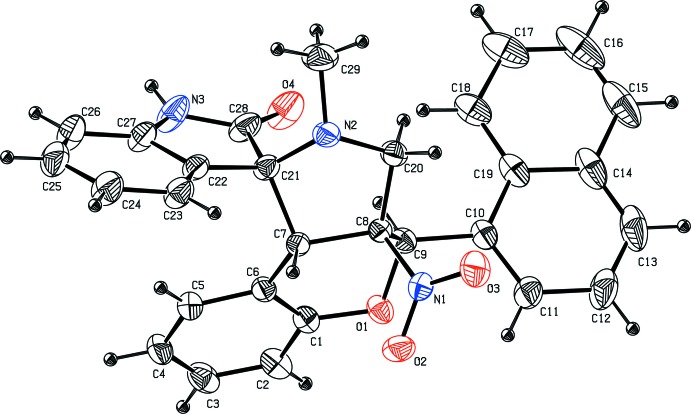
The molecular structure of the title molecule, with atom labelling. Displacement ellipsoids are drawn at the 30% probability level.

**Fig. 2.**
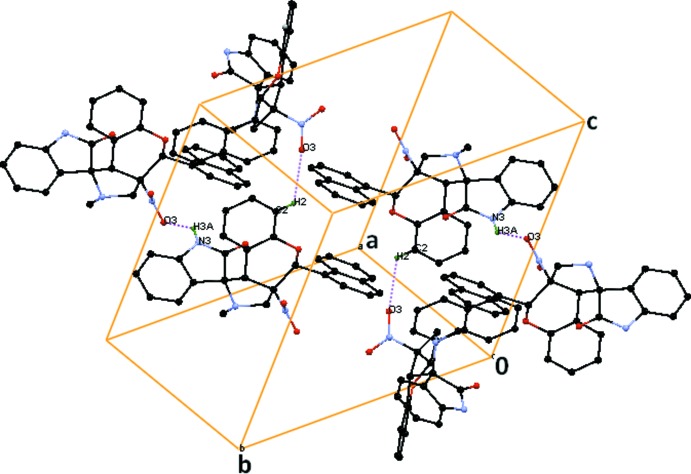
A partial view, ca. perpendicular to (110), of the crystal packing of the title compound. The hydrogen bonds are shown as dashed lines - see Table 1 for details; H-atoms not involved in hydrogen bonding have been omitted for clarity.

## supplementary crystallographic information

### Comment

4H-chromenes are biologically important compounds used as synthetic ligands in the design of drugs and discovery processes. They exhibit numerous biological and pharmacological properties, such as anti-viral, anti-fungal, antiinflammatory, anti- diabetic, cardionthonic, anti anaphylactic and anti-cancer (Cai, 2008, 2007; Cai *et al.*, 2006; Gabor, 1988; Brooks, 1998; Valenti *et al.*, 1993; Hyana & Saimoto, 1987; Tang *et al.*, 2007). Indoline-2-one and its derivatives have been used as precursors to synthesis pharmaceuticals (Colgan *et al.*, 1996). Continuing our interest in such compounds we have synthesized the title compound and report herein on its crystal structure.

In the title compound, Fig. 1, the pyrrole ring (N2/C7/C8/C20/C21) adopts a *twist* conformation on bond C21-N2, while the pyran ring (O1/C1/C6-C9) adopts a *envelope* conformation with atom C9 as the flap. The pyrrole ring (N2/C7/C8/C20/C21) mean plane makes a dihedral angle of 89.73 (8)° with the mean plane of the indoline-2-one ring system (N3/C21-C28), which shows that they are almost orthogonal to each other. The same pyrrole ring mean plane makes dihedral angles of 47.80 (8) Å with the naphthalene ring system (C10-C19) and 32.38 (8)° with the pyran ring mean plane (O1/C1/C6-C9), and the oxygen atom O4 attached to the pyrrole ring deviates by -0.0886 (2) Å. The nitro group (N1/O2/O3) is inclined to the mean plane of the pyrrole ring, to which it is attached, with a dihedral angle of 50.76 (19) °.

In the crystal, adjacent molecules are linked via N—H···O hydrogen bonds forming chains propagating along [100]; see Table 1 and Fig. 2. The chains are linked via weak C-H···O hydrogen bonds forming two-dimensional networks, lying parallel to (101), and consolidated by C-H···π interactions (Table 1).

### Experimental

To a solution of isatin (1 equiv) and sarcosine (1.4 equiv) in dry toluene, was added 2-(naphthalen-1-yl)-3-nitro-2H-chromene (1 equiv) under a nitrogen atmosphere. The reaction mixture was refluxed for 24h in a Dean-Stark apparatus to give the cycloadducts. After completion of the reaction as indicated by TLC, the solvent was evaporated under reduced pressure. The crude product was extracted with dichloromethane. The organic layer was dried with anhydrous sodium sulphate and concentrated in *vacuo*. The crude product obtained was purified by column chromatography using hexane/EtOAc (7:3) as eluent. Single crystals suitable for X-ray diffraction were obtained by slow evaporation of a solution of the title compound in ethyl acetate at room temperature.

### Refinement

The NH H atom was located in a difference Fourier map and refined with a distance restraint of N-H = 0.88 (1) Å with U_iso_(H) = 1.2U_eq_(N). The C-bound H atoms were placed in calculated positions and treated as riding atoms: C—H = 0.93 - 0.97 Å, with U_iso_(H) = 1.5U_eq_(C) for methyl H atoms and = 1.2U_eq_(C) for other H atoms.

### Crystal data

**Table:**

C_29_H_23_N_3_O_4_	*F*(000) = 1000
*M**_r_* = 477.50	*D*_x_ = 1.348 Mg m^−^^3^
Monoclinic, *P*2_1_/*n*	Mo *K*α radiation, λ = 0.71073 Å
Hall symbol: -P 2yn	Cell parameters from 5856 reflections
*a* = 9.4359 (6) Å	θ = 1.8–28.3°
*b* = 16.5086 (11) Å	µ = 0.09 mm^−^^1^
*c* = 15.1964 (10) Å	*T* = 293 K
β = 96.363 (4)°	Block, colourless
*V* = 2352.6 (3) Å^3^	0.30 × 0.25 × 0.20 mm
*Z* = 4	

### Data collection

**Table:**

Bruker SMART APEXII area-detector diffractometer	5856 independent reflections
Radiation source: fine-focus sealed tube	3862 reflections with *I* > 2σ(*I*)
Graphite monochromator	*R*_int_ = 0.035
ω and φ scans	θ_max_ = 28.3°, θ_min_ = 1.8°
Absorption correction: multi-scan (*SADABS*; Bruker, 2008)	*h* = −12→12
*T*_min_ = 0.973, *T*_max_ = 0.982	*k* = −22→22
22349 measured reflections	*l* = −14→20

### Refinement

**Table:**

Refinement on *F*^2^	Primary atom site location: structure-invariant direct methods
Least-squares matrix: full	Secondary atom site location: difference Fourier map
*R*[*F*^2^ > 2σ(*F*^2^)] = 0.048	Hydrogen site location: inferred from neighbouring sites
*wR*(*F*^2^) = 0.141	H atoms treated by a mixture of independent and constrained refinement
*S* = 1.03	*w* = 1/[σ^2^(*F*_o_^2^) + (0.0641*P*)^2^ + 0.4707*P*] where *P* = (*F*_o_^2^ + 2*F*_c_^2^)/3
5856 reflections	(Δ/σ)_max_ < 0.001
329 parameters	Δρ_max_ = 0.27 e Å^−^^3^
1 restraint	Δρ_min_ = −0.18 e Å^−^^3^

### Special details

**Table:**

Geometry. All esds (except the esd in the dihedral angle between two l.s. planes) are estimated using the full covariance matrix. The cell esds are taken into account individually in the estimation of esds in distances, angles and torsion angles; correlations between esds in cell parameters are only used when they are defined by crystal symmetry. An approximate (isotropic) treatment of cell esds is used for estimating esds involving l.s. planes.
Refinement. Refinement of F^2^ against ALL reflections. The weighted R-factor wR and goodness of fit S are based on F^2^, conventional R-factors R are based on F, with F set to zero for negative F^2^. The threshold expression of F^2^ > 2sigma(F^2^) is used only for calculating R-factors(gt) etc. and is not relevant to the choice of reflections for refinement. R-factors based on F^2^ are statistically about twice as large as those based on F, and R- factors based on ALL data will be even larger.

### Fractional atomic coordinates and isotropic or equivalent isotropic displacement parameters (Å^2^)

**Table:**

	*x*	*y*	*z*	*U*_iso_*/*U*_eq_	
C1	0.48112 (16)	0.19305 (10)	0.41572 (11)	0.0398 (4)	
C2	0.44179 (19)	0.19055 (11)	0.32492 (11)	0.0499 (4)	
H2	0.4791	0.2279	0.2879	0.060*	
C3	0.3479 (2)	0.13283 (12)	0.29034 (13)	0.0568 (5)	
H3	0.3204	0.1314	0.2297	0.068*	
C4	0.29370 (19)	0.07669 (13)	0.34465 (13)	0.0587 (5)	
H4	0.2300	0.0375	0.3206	0.070*	
C5	0.33401 (17)	0.07866 (12)	0.43511 (12)	0.0510 (4)	
H5	0.2986	0.0399	0.4714	0.061*	
C6	0.42704 (15)	0.13804 (10)	0.47241 (11)	0.0389 (4)	
C7	0.47639 (14)	0.13939 (9)	0.57035 (10)	0.0344 (3)	
H7	0.5304	0.0893	0.5836	0.041*	
C8	0.57755 (15)	0.21006 (9)	0.59985 (10)	0.0336 (3)	
C9	0.56344 (17)	0.28055 (9)	0.53218 (10)	0.0379 (3)	
H9	0.4669	0.3027	0.5312	0.046*	
C10	0.66706 (18)	0.34982 (10)	0.55074 (10)	0.0436 (4)	
C11	0.8044 (2)	0.34132 (13)	0.53052 (12)	0.0574 (5)	
H11	0.8309	0.2936	0.5042	0.069*	
C12	0.9055 (3)	0.40300 (18)	0.54864 (15)	0.0809 (7)	
H12	0.9988	0.3955	0.5359	0.097*	
C13	0.8670 (3)	0.47382 (17)	0.58491 (16)	0.0879 (9)	
H13	0.9349	0.5142	0.5974	0.105*	
C14	0.7272 (3)	0.48697 (12)	0.60376 (13)	0.0698 (6)	
C15	0.6841 (5)	0.56123 (15)	0.64053 (17)	0.0970 (10)	
H15	0.7512	0.6020	0.6531	0.116*	
C16	0.5499 (5)	0.57387 (15)	0.65736 (19)	0.1058 (11)	
H16	0.5250	0.6231	0.6813	0.127*	
C17	0.4470 (4)	0.51399 (14)	0.63942 (16)	0.0844 (8)	
H17	0.3537	0.5237	0.6507	0.101*	
C18	0.4821 (3)	0.44085 (11)	0.60523 (13)	0.0609 (5)	
H18	0.4123	0.4013	0.5940	0.073*	
C19	0.6225 (2)	0.42464 (10)	0.58675 (11)	0.0506 (4)	
C20	0.54002 (18)	0.23565 (10)	0.69127 (11)	0.0446 (4)	
H20A	0.6250	0.2399	0.7332	0.054*	
H20B	0.4906	0.2873	0.6883	0.054*	
C21	0.36114 (16)	0.14308 (9)	0.63575 (11)	0.0403 (4)	
C22	0.28572 (16)	0.06403 (10)	0.64729 (11)	0.0430 (4)	
C23	0.33684 (19)	−0.00967 (11)	0.67741 (14)	0.0554 (5)	
H23	0.4329	−0.0164	0.6974	0.066*	
C24	0.2425 (2)	−0.07411 (12)	0.67744 (16)	0.0686 (6)	
H24	0.2756	−0.1245	0.6980	0.082*	
C25	0.1010 (2)	−0.06438 (13)	0.64754 (17)	0.0713 (6)	
H25	0.0397	−0.1085	0.6474	0.086*	
C26	0.0477 (2)	0.00972 (14)	0.61763 (16)	0.0665 (6)	
H26	−0.0482	0.0164	0.5973	0.080*	
C27	0.14211 (17)	0.07308 (11)	0.61905 (13)	0.0525 (5)	
C28	0.23476 (18)	0.19965 (12)	0.60220 (14)	0.0554 (5)	
C29	0.3701 (3)	0.19088 (14)	0.79120 (15)	0.0748 (7)	
H29A	0.3039	0.2339	0.7748	0.112*	
H29B	0.4366	0.2080	0.8401	0.112*	
H29C	0.3192	0.1441	0.8081	0.112*	
N1	0.72963 (13)	0.17834 (8)	0.60492 (9)	0.0404 (3)	
N2	0.44715 (15)	0.17065 (8)	0.71623 (9)	0.0451 (3)	
N3	0.11545 (15)	0.15370 (11)	0.59269 (13)	0.0669 (5)	
H3A	0.0310 (14)	0.1754 (13)	0.5819 (15)	0.080*	
O1	0.57898 (12)	0.25128 (7)	0.44517 (7)	0.0437 (3)	
O2	0.75903 (12)	0.13501 (8)	0.54490 (9)	0.0585 (4)	
O3	0.81715 (14)	0.20033 (10)	0.66412 (10)	0.0701 (4)	
O4	0.24095 (15)	0.27211 (9)	0.58777 (12)	0.0780 (5)	

### Atomic displacement parameters (Å^2^)

**Table:**

	*U*^11^	*U*^22^	*U*^33^	*U*^12^	*U*^13^	*U*^23^
C1	0.0412 (8)	0.0353 (8)	0.0413 (9)	0.0053 (7)	−0.0030 (6)	−0.0016 (7)
C2	0.0618 (11)	0.0434 (10)	0.0418 (9)	0.0090 (8)	−0.0057 (8)	0.0012 (8)
C3	0.0593 (11)	0.0611 (12)	0.0460 (10)	0.0100 (10)	−0.0116 (8)	−0.0120 (9)
C4	0.0464 (9)	0.0657 (13)	0.0619 (12)	−0.0070 (9)	−0.0040 (8)	−0.0248 (10)
C5	0.0434 (8)	0.0531 (11)	0.0569 (11)	−0.0079 (8)	0.0074 (8)	−0.0123 (9)
C6	0.0338 (7)	0.0381 (8)	0.0441 (9)	0.0032 (6)	0.0006 (6)	−0.0069 (7)
C7	0.0323 (7)	0.0292 (7)	0.0421 (8)	0.0012 (6)	0.0061 (6)	−0.0014 (6)
C8	0.0342 (7)	0.0323 (7)	0.0347 (7)	−0.0004 (6)	0.0050 (6)	−0.0010 (6)
C9	0.0444 (8)	0.0334 (8)	0.0361 (8)	−0.0005 (6)	0.0048 (6)	−0.0006 (6)
C10	0.0598 (10)	0.0374 (9)	0.0333 (8)	−0.0099 (8)	0.0039 (7)	0.0049 (7)
C11	0.0649 (11)	0.0593 (12)	0.0501 (10)	−0.0193 (9)	0.0156 (9)	0.0055 (9)
C12	0.0780 (14)	0.098 (2)	0.0683 (14)	−0.0452 (14)	0.0160 (11)	0.0106 (14)
C13	0.122 (2)	0.0761 (17)	0.0643 (14)	−0.0620 (17)	0.0051 (14)	0.0065 (13)
C14	0.1206 (19)	0.0442 (11)	0.0417 (10)	−0.0297 (12)	−0.0040 (11)	0.0094 (9)
C15	0.182 (3)	0.0418 (14)	0.0624 (15)	−0.0313 (18)	−0.0059 (18)	−0.0028 (11)
C16	0.209 (4)	0.0352 (13)	0.0693 (17)	0.0116 (19)	−0.004 (2)	−0.0054 (11)
C17	0.144 (2)	0.0465 (13)	0.0619 (14)	0.0291 (14)	0.0072 (14)	0.0016 (11)
C18	0.0948 (15)	0.0369 (10)	0.0502 (11)	0.0115 (10)	0.0044 (10)	0.0026 (8)
C19	0.0856 (13)	0.0325 (9)	0.0323 (8)	−0.0086 (9)	−0.0001 (8)	0.0070 (7)
C20	0.0548 (9)	0.0393 (9)	0.0418 (9)	−0.0063 (7)	0.0141 (7)	−0.0039 (7)
C21	0.0372 (7)	0.0332 (8)	0.0525 (10)	0.0020 (6)	0.0130 (7)	0.0025 (7)
C22	0.0388 (8)	0.0383 (9)	0.0543 (10)	−0.0007 (7)	0.0160 (7)	0.0016 (7)
C23	0.0460 (9)	0.0426 (10)	0.0796 (13)	0.0021 (8)	0.0165 (9)	0.0083 (9)
C24	0.0641 (12)	0.0389 (10)	0.1073 (18)	−0.0027 (9)	0.0289 (12)	0.0067 (11)
C25	0.0616 (12)	0.0537 (13)	0.1026 (18)	−0.0186 (10)	0.0268 (12)	−0.0024 (12)
C26	0.0435 (9)	0.0698 (15)	0.0872 (15)	−0.0114 (9)	0.0115 (9)	0.0063 (12)
C27	0.0388 (8)	0.0523 (11)	0.0687 (12)	−0.0016 (8)	0.0162 (8)	0.0074 (9)
C28	0.0435 (9)	0.0467 (10)	0.0795 (13)	0.0098 (8)	0.0225 (9)	0.0116 (10)
C29	0.0996 (16)	0.0645 (13)	0.0693 (13)	−0.0159 (12)	0.0498 (12)	−0.0099 (11)
N1	0.0365 (6)	0.0426 (8)	0.0416 (7)	−0.0025 (6)	0.0017 (6)	0.0047 (6)
N2	0.0553 (8)	0.0386 (8)	0.0443 (8)	−0.0062 (6)	0.0185 (6)	−0.0018 (6)
N3	0.0339 (7)	0.0602 (11)	0.1080 (14)	0.0085 (7)	0.0141 (8)	0.0250 (10)
O1	0.0572 (7)	0.0395 (6)	0.0337 (6)	−0.0071 (5)	0.0024 (5)	−0.0003 (5)
O2	0.0446 (6)	0.0695 (9)	0.0625 (8)	0.0110 (6)	0.0109 (6)	−0.0121 (7)
O3	0.0476 (7)	0.0900 (11)	0.0677 (9)	−0.0019 (7)	−0.0162 (6)	−0.0144 (8)
O4	0.0572 (8)	0.0453 (8)	0.1352 (14)	0.0152 (6)	0.0277 (8)	0.0235 (8)

### Geometric parameters (Å, º)

**Table:**

C1—O1	1.3731 (19)	C16—C17	1.391 (4)
C1—C6	1.387 (2)	C16—H16	0.9300
C1—C2	1.389 (2)	C17—C18	1.369 (3)
C2—C3	1.366 (3)	C17—H17	0.9300
C2—H2	0.9300	C18—C19	1.410 (3)
C3—C4	1.377 (3)	C18—H18	0.9300
C3—H3	0.9300	C20—N2	1.462 (2)
C4—C5	1.385 (3)	C20—H20A	0.9700
C4—H4	0.9300	C20—H20B	0.9700
C5—C6	1.394 (2)	C21—N2	1.464 (2)
C5—H5	0.9300	C21—C22	1.506 (2)
C6—C7	1.510 (2)	C21—C28	1.555 (2)
C7—C8	1.542 (2)	C22—C23	1.369 (2)
C7—C21	1.5535 (19)	C22—C27	1.383 (2)
C7—H7	0.9800	C23—C24	1.387 (3)
C8—N1	1.5215 (19)	C23—H23	0.9300
C8—C20	1.531 (2)	C24—C25	1.371 (3)
C8—C9	1.549 (2)	C24—H24	0.9300
C9—O1	1.4304 (18)	C25—C26	1.380 (3)
C9—C10	1.511 (2)	C25—H25	0.9300
C9—H9	0.9800	C26—C27	1.372 (3)
C10—C11	1.371 (3)	C26—H26	0.9300
C10—C19	1.433 (2)	C27—N3	1.405 (2)
C11—C12	1.402 (3)	C28—O4	1.219 (2)
C11—H11	0.9300	C28—N3	1.352 (2)
C12—C13	1.359 (4)	C29—N2	1.456 (2)
C12—H12	0.9300	C29—H29A	0.9600
C13—C14	1.398 (4)	C29—H29B	0.9600
C13—H13	0.9300	C29—H29C	0.9600
C14—C15	1.425 (4)	N1—O3	1.2077 (18)
C14—C19	1.430 (3)	N1—O2	1.2151 (17)
C15—C16	1.336 (5)	N3—H3A	0.872 (9)
C15—H15	0.9300		
			
O1—C1—C6	122.43 (14)	C18—C17—H17	119.7
O1—C1—C2	116.13 (15)	C16—C17—H17	119.7
C6—C1—C2	121.41 (15)	C17—C18—C19	121.0 (2)
C3—C2—C1	119.56 (18)	C17—C18—H18	119.5
C3—C2—H2	120.2	C19—C18—H18	119.5
C1—C2—H2	120.2	C18—C19—C14	118.22 (19)
C2—C3—C4	120.48 (17)	C18—C19—C10	124.17 (17)
C2—C3—H3	119.8	C14—C19—C10	117.60 (19)
C4—C3—H3	119.8	N2—C20—C8	103.81 (12)
C3—C4—C5	119.97 (17)	N2—C20—H20A	111.0
C3—C4—H4	120.0	C8—C20—H20A	111.0
C5—C4—H4	120.0	N2—C20—H20B	111.0
C4—C5—C6	120.76 (18)	C8—C20—H20B	111.0
C4—C5—H5	119.6	H20A—C20—H20B	109.0
C6—C5—H5	119.6	N2—C21—C22	113.42 (14)
C1—C6—C5	117.79 (15)	N2—C21—C7	100.61 (12)
C1—C6—C7	120.54 (13)	C22—C21—C7	114.45 (13)
C5—C6—C7	121.53 (15)	N2—C21—C28	114.91 (14)
C6—C7—C8	114.27 (12)	C22—C21—C28	101.74 (13)
C6—C7—C21	118.03 (12)	C7—C21—C28	112.30 (13)
C8—C7—C21	103.83 (12)	C23—C22—C27	119.77 (16)
C6—C7—H7	106.7	C23—C22—C21	130.97 (15)
C8—C7—H7	106.7	C27—C22—C21	109.24 (15)
C21—C7—H7	106.7	C22—C23—C24	118.64 (18)
N1—C8—C20	111.16 (12)	C22—C23—H23	120.7
N1—C8—C7	107.71 (12)	C24—C23—H23	120.7
C20—C8—C7	105.94 (11)	C25—C24—C23	120.8 (2)
N1—C8—C9	107.66 (11)	C25—C24—H24	119.6
C20—C8—C9	112.57 (12)	C23—C24—H24	119.6
C7—C8—C9	111.74 (12)	C24—C25—C26	121.22 (19)
O1—C9—C10	107.32 (12)	C24—C25—H25	119.4
O1—C9—C8	110.48 (12)	C26—C25—H25	119.4
C10—C9—C8	116.01 (13)	C27—C26—C25	117.29 (18)
O1—C9—H9	107.6	C27—C26—H26	121.4
C10—C9—H9	107.6	C25—C26—H26	121.4
C8—C9—H9	107.6	C26—C27—C22	122.29 (18)
C11—C10—C19	119.88 (16)	C26—C27—N3	128.49 (17)
C11—C10—C9	119.25 (16)	C22—C27—N3	109.22 (15)
C19—C10—C9	120.85 (15)	O4—C28—N3	125.97 (17)
C10—C11—C12	121.4 (2)	O4—C28—C21	126.73 (16)
C10—C11—H11	119.3	N3—C28—C21	107.29 (15)
C12—C11—H11	119.3	N2—C29—H29A	109.5
C13—C12—C11	119.8 (2)	N2—C29—H29B	109.5
C13—C12—H12	120.1	H29A—C29—H29B	109.5
C11—C12—H12	120.1	N2—C29—H29C	109.5
C12—C13—C14	121.2 (2)	H29A—C29—H29C	109.5
C12—C13—H13	119.4	H29B—C29—H29C	109.5
C14—C13—H13	119.4	O3—N1—O2	122.82 (14)
C13—C14—C15	122.1 (2)	O3—N1—C8	119.83 (14)
C13—C14—C19	120.0 (2)	O2—N1—C8	117.20 (13)
C15—C14—C19	117.9 (3)	C29—N2—C20	113.58 (14)
C16—C15—C14	121.8 (3)	C29—N2—C21	116.52 (15)
C16—C15—H15	119.1	C20—N2—C21	107.71 (12)
C14—C15—H15	119.1	C28—N3—C27	112.36 (15)
C15—C16—C17	120.5 (3)	C28—N3—H3A	121.5 (15)
C15—C16—H16	119.8	C27—N3—H3A	125.0 (15)
C17—C16—H16	119.8	C1—O1—C9	113.80 (12)
C18—C17—C16	120.6 (3)		
			
O1—C1—C2—C3	178.19 (15)	C7—C8—C20—N2	11.54 (16)
C6—C1—C2—C3	0.0 (2)	C9—C8—C20—N2	133.94 (13)
C1—C2—C3—C4	−0.8 (3)	C6—C7—C21—N2	−161.75 (13)
C2—C3—C4—C5	0.2 (3)	C8—C7—C21—N2	−34.11 (14)
C3—C4—C5—C6	1.3 (3)	C6—C7—C21—C22	76.29 (18)
O1—C1—C6—C5	−176.63 (14)	C8—C7—C21—C22	−156.07 (13)
C2—C1—C6—C5	1.5 (2)	C6—C7—C21—C28	−39.06 (19)
O1—C1—C6—C7	−0.9 (2)	C8—C7—C21—C28	88.58 (16)
C2—C1—C6—C7	177.16 (14)	N2—C21—C22—C23	−54.1 (2)
C4—C5—C6—C1	−2.1 (2)	C7—C21—C22—C23	60.5 (3)
C4—C5—C6—C7	−177.75 (15)	C28—C21—C22—C23	−178.11 (19)
C1—C6—C7—C8	5.17 (19)	N2—C21—C22—C27	127.56 (15)
C5—C6—C7—C8	−179.29 (14)	C7—C21—C22—C27	−117.77 (16)
C1—C6—C7—C21	127.66 (15)	C28—C21—C22—C27	3.58 (18)
C5—C6—C7—C21	−56.8 (2)	C27—C22—C23—C24	1.0 (3)
C6—C7—C8—N1	−97.00 (14)	C21—C22—C23—C24	−177.17 (18)
C21—C7—C8—N1	133.06 (12)	C22—C23—C24—C25	0.3 (3)
C6—C7—C8—C20	143.98 (13)	C23—C24—C25—C26	−0.8 (4)
C21—C7—C8—C20	14.04 (15)	C24—C25—C26—C27	0.0 (3)
C6—C7—C8—C9	21.05 (16)	C25—C26—C27—C22	1.3 (3)
C21—C7—C8—C9	−108.89 (13)	C25—C26—C27—N3	−179.9 (2)
N1—C8—C9—O1	65.62 (15)	C23—C22—C27—C26	−1.9 (3)
C20—C8—C9—O1	−171.52 (12)	C21—C22—C27—C26	176.67 (18)
C7—C8—C9—O1	−52.45 (15)	C23—C22—C27—N3	179.16 (17)
N1—C8—C9—C10	−56.78 (16)	C21—C22—C27—N3	−2.3 (2)
C20—C8—C9—C10	66.07 (17)	N2—C21—C28—O4	52.8 (3)
C7—C8—C9—C10	−174.86 (12)	C22—C21—C28—O4	175.7 (2)
O1—C9—C10—C11	−45.55 (19)	C7—C21—C28—O4	−61.4 (3)
C8—C9—C10—C11	78.51 (18)	N2—C21—C28—N3	−126.64 (16)
O1—C9—C10—C19	132.72 (15)	C22—C21—C28—N3	−3.67 (19)
C8—C9—C10—C19	−103.22 (17)	C7—C21—C28—N3	119.16 (17)
C19—C10—C11—C12	3.4 (3)	C20—C8—N1—O3	−25.4 (2)
C9—C10—C11—C12	−178.27 (17)	C7—C8—N1—O3	−141.06 (15)
C10—C11—C12—C13	−1.7 (3)	C9—C8—N1—O3	98.29 (16)
C11—C12—C13—C14	−0.7 (4)	C20—C8—N1—O2	158.88 (14)
C12—C13—C14—C15	−179.0 (2)	C7—C8—N1—O2	43.25 (17)
C12—C13—C14—C19	1.4 (3)	C9—C8—N1—O2	−77.40 (17)
C13—C14—C15—C16	179.1 (3)	C8—C20—N2—C29	−165.99 (17)
C19—C14—C15—C16	−1.3 (4)	C8—C20—N2—C21	−35.37 (16)
C14—C15—C16—C17	0.1 (4)	C22—C21—N2—C29	−64.53 (19)
C15—C16—C17—C18	0.8 (4)	C7—C21—N2—C29	172.78 (15)
C16—C17—C18—C19	−0.5 (3)	C28—C21—N2—C29	51.9 (2)
C17—C18—C19—C14	−0.7 (3)	C22—C21—N2—C20	166.51 (12)
C17—C18—C19—C10	−179.78 (18)	C7—C21—N2—C20	43.82 (15)
C13—C14—C19—C18	−178.90 (19)	C28—C21—N2—C20	−77.02 (16)
C15—C14—C19—C18	1.5 (3)	O4—C28—N3—C27	−176.8 (2)
C13—C14—C19—C10	0.3 (3)	C21—C28—N3—C27	2.6 (2)
C15—C14—C19—C10	−179.30 (17)	C26—C27—N3—C28	−179.2 (2)
C11—C10—C19—C18	176.46 (17)	C22—C27—N3—C28	−0.2 (2)
C9—C10—C19—C18	−1.8 (2)	C6—C1—O1—C9	−32.43 (19)
C11—C10—C19—C14	−2.7 (2)	C2—C1—O1—C9	149.38 (14)
C9—C10—C19—C14	179.08 (15)	C10—C9—O1—C1	−173.96 (13)
N1—C8—C20—N2	−105.18 (14)	C8—C9—O1—C1	58.67 (16)

### Hydrogen-bond geometry (Å, º)

Cg1 is the centroid of the C10–C14/C19 ring.

**Table:**

*D*—H···*A*	*D*—H	H···*A*	*D*···*A*	*D*—H···*A*
C9—H9···O4	0.98	2.44	3.250 (2)	140
N3—H3*A*···O3^i^	0.87 (2)	2.52 (2)	3.220 (2)	138 (2)
C2—H2···O3^ii^	0.93	2.58	3.156 (2)	121
C3—H3···*Cg*1^ii^	0.93	2.57	3.473 (2)	164

Symmetry codes: (i) *x*−1, *y*, *z*; (ii) *x*−1/2, −*y*+1/2, *z*−1/2.
